# 4-[(*E*)-(4-Fluoro­benzyl­idene)amino]-3-methyl-1*H*-1,2,4-triazole-5(4*H*)-thione

**DOI:** 10.1107/S1600536812019174

**Published:** 2012-05-05

**Authors:** H. C. Devarajegowda, S. Jeyaseelan, R. Sathishkumar, Agnes Sylvia D’souza, Alphonsus D’souza

**Affiliations:** a Department of Physics, Yuvaraja’s College (Constituent College), University of Mysore, Mysore 570 005, Karnataka, India; b Solid State and Structural Chemistry Unit, Indian Institute of Science, Bangalore, Karnataka, India; c Department of Chemistry, St. Philomena’s College, Mysore 570 015, Karnataka, India

## Abstract

In the asymmetric unit of the title compound, C_10_H_9_FN_4_S, there are two independent mol­ecules in which the dihedral angles between the 1,2,4-triazole and benzene rings are 36.85 (10) and 7.81 (10)°. In the crystal, N—H⋯S inter­actions link pairs of independent mol­ecules into dimers. There are also π–π inter­actions between the triazole and benzene rings of inversion-related pairs of the more planar mol­ecule [centroid–centroid distance = 3.6430 (13) Å].

## Related literature

For background information on the properties and uses of chalcone derivatives, see: Temple (1981 ▶); Holla *et al.* (1998 ▶); Heidelberger *et al.* (1957 ▶); Andersson & MacGowan (2003 ▶). For a related structure, see: Devarajegowda *et al.* (2010 ▶).
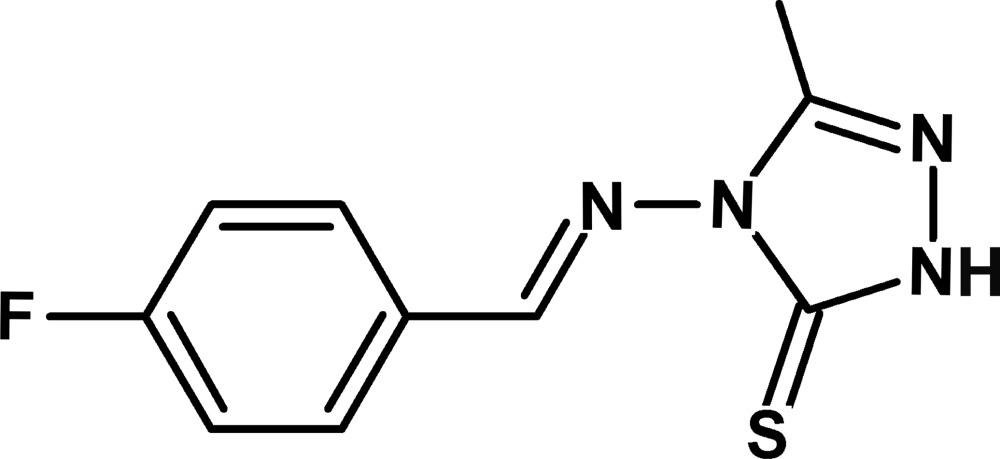


## Experimental

### 

#### Crystal data

C_10_H_9_FN_4_S
*M* *_r_* = 236.27
Triclinic, 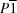

*a* = 9.0048 (19) Å
*b* = 10.811 (2) Å
*c* = 12.729 (3) Å
α = 101.205 (3)°
β = 103.899 (3)°
γ = 112.376 (3)°
*V* = 1054.4 (4) Å^3^
*Z* = 4
Mo *K*α radiation
μ = 0.30 mm^−1^
*T* = 293 K
0.52 × 0.24 × 0.13 mm

#### Data collection

Bruker SMART CCD area-detector diffractometer
Absorption correction: multi-scan (*SADABS*; Sheldrick, 2007 ▶) *T*_min_ = 0.77, *T*_max_ = 1.00
9923 measured reflections
3698 independent reflections
3383 reflections with *I* > 2σ(*I*)
*R*_int_ = 0.017

#### Refinement

*R*[*F* ^2^ > 2σ(*F* ^2^)] = 0.033
*wR*(*F* ^2^) = 0.085
*S* = 1.05
3698 reflections
291 parameters
H-atom parameters constrained
Δρ_max_ = 0.30 e Å^−3^
Δρ_min_ = −0.28 e Å^−3^

### 

Data collection: *SMART* (Bruker, 2001 ▶); cell refinement: *SAINT* (Bruker, 2001 ▶); data reduction: *SAINT*; program(s) used to solve structure: *SHELXS97* (Sheldrick, 2008 ▶); program(s) used to refine structure: *SHELXL97* (Sheldrick, 2008 ▶); molecular graphics: *ORTEP-3* (Farrugia, 1997 ▶); software used to prepare material for publication: *SHELXL97*.

## Supplementary Material

Crystal structure: contains datablock(s) I, global. DOI: 10.1107/S1600536812019174/pk2403sup1.cif

Structure factors: contains datablock(s) I. DOI: 10.1107/S1600536812019174/pk2403Isup2.hkl

Supplementary material file. DOI: 10.1107/S1600536812019174/pk2403Isup3.cml

Additional supplementary materials:  crystallographic information; 3D view; checkCIF report

## Figures and Tables

**Table 1:** Hydrogen-bond geometry (Å, °)

*D*—H⋯*A*	*D*—H	H⋯*A*	*D*⋯*A*	*D*—H⋯*A*
N3*A*—H3*A*⋯S1*B*	0.86	2.45	3.2840 (18)	164
N3*B*—H3*B*⋯S1*A*	0.86	2.51	3.3691 (18)	172

**Fig. 1.**
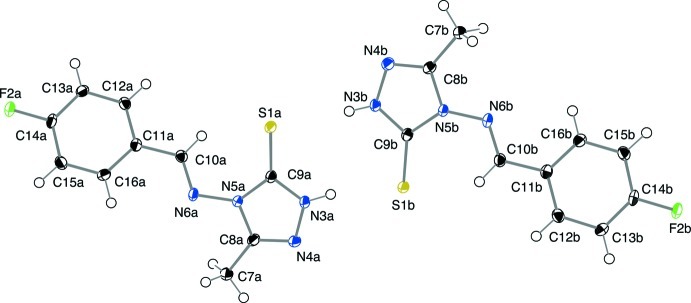
The molecular structure of the title compound. Displacement ellipsoids are drawn at the 50% probability level.

**Fig. 2.**
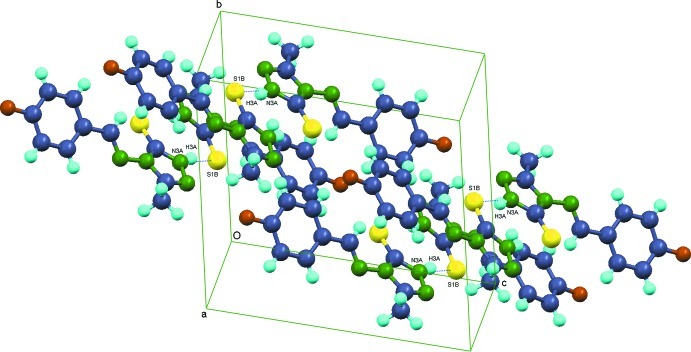
The packing of the molecules showing the formation of hydrogen bonds that link inequivalent molecules into dimers via N3A—H3A···S1B and N3A—H3A···S1B.

## supplementary crystallographic information

### Comment

4-Amino-5-mercapto-1,2,4-triazoles are the starting materials for the synthesis of a wide variety of heterocyclic derivatives which are of great importance in medicinal chemistry (Temple, 1981). Many Schiff & Mannich bases derived from 1,2,4-triazoles possess protozoacidal and bactericidal activities (Holla *et al.*, 1998). Furthermore, fluorinated heterocycles have been shown to exhibit a wide variety of biocidal activities. Compounds such as fluorouracil and fluoroquinolone *etc*. have been used as anticancer agents and antibiotics respectively (Heidelberger *et al.*, 1957; Andersson & MacGowan, 2003).

The asymmetric unit of crystals of 4-{[(1E)-(4-fluorophenyl)methylene] amino}-5-methyl-2,4-dihydro-3*H*-1,2,4-triazole-3-thione, C_10_H_9_F N_4_S, contain two crystallographically independent molecules (Fig. 1). The 1,2,4 triazole rings (N3A,N4A,N5A,C8A,C9A and (N3B,N4B,N5B,C8B,C9B) are not coplanar with their respective benzene ring (C11A—C16A) and (C11B—C16B) systems; the dihedral angle between the two planes being 36.85 (10)° and 7.81 (10)° in the two molecules. In the crystal, N3A—H3A···S1B and N3B—H3B···S1A interactions link pairs of inequivalent molecules into dimers (Table 1.). Finally, π-π interactions between inversion-related pairs of the more planar molecule occur between the triazole (N3B,N4B,N5B,C8B,C9B) and benzene (C11B—C16B) rings [centroid-centroid distance = 3.6430 (13) Å], which stabilize the crystal packing (Fig. 2).

### Experimental

An equimolar mixture of the triazole (1; 0.02 mol) and 4-fluorobenzaldehyde (0.02 mol) in absolute ethanol (30 ml) was refluxed with concentrated H_2_SO_4_ (0.5 ml) for 1–2 hrs. On cooling the reaction mixture, the solid product was separated and re-crystallized using ethanol as solvent.

### Refinement

All H atoms were placed at calculated positions and refined as riding, N—H = 0.86, C*sp*^2^—H = 0.93 Å and *C*(methyl)—H = 0.96 Å. *U*_iso_(H) = *xU*_eq_(C,*N*), where *x* = 1.5 for methyl H and 1.2 for all other H atoms.

### Crystal data

**Table:**

C_10_H_9_FN_4_S	*Z* = 4
*M**_r_* = 236.27	*F*(000) = 488
Triclinic, *P*1	*D*_x_ = 1.488 Mg m^−^^3^
Hall symbol: -P 1	Melting point: 441 K
*a* = 9.0048 (19) Å	Mo *K*α radiation, λ = 0.71073 Å
*b* = 10.811 (2) Å	Cell parameters from 3698 reflections
*c* = 12.729 (3) Å	θ = 1.7–25.0°
α = 101.205 (3)°	µ = 0.30 mm^−^^1^
β = 103.899 (3)°	*T* = 293 K
γ = 112.376 (3)°	Plate, colourless
*V* = 1054.4 (4) Å^3^	0.52 × 0.24 × 0.13 mm

### Data collection

**Table:**

Bruker SMART CCD area-detector diffractometer	3698 independent reflections
Radiation source: fine-focus sealed tube	3383 reflections with *I* > 2σ(*I*)
Graphite monochromator	*R*_int_ = 0.017
ω and φ scans	θ_max_ = 25.0°, θ_min_ = 1.7°
Absorption correction: multi-scan (*SADABS*; Sheldrick, 2007)	*h* = −10→10
*T*_min_ = 0.77, *T*_max_ = 1.00	*k* = −12→12
9923 measured reflections	*l* = −15→15

### Refinement

**Table:**

Refinement on *F*^2^	Primary atom site location: structure-invariant direct methods
Least-squares matrix: full	Secondary atom site location: difference Fourier map
*R*[*F*^2^ > 2σ(*F*^2^)] = 0.033	Hydrogen site location: inferred from neighbouring sites
*wR*(*F*^2^) = 0.085	H-atom parameters constrained
*S* = 1.05	*w* = 1/[σ^2^(*F*_o_^2^) + (0.0428*P*)^2^ + 0.5511*P*] where *P* = (*F*_o_^2^ + 2*F*_c_^2^)/3
3698 reflections	(Δ/σ)_max_ = 0.001
291 parameters	Δρ_max_ = 0.30 e Å^−^^3^
0 restraints	Δρ_min_ = −0.28 e Å^−^^3^

### Special details

**Table:**

Geometry. All s.u.'s (except the s.u. in the dihedral angle between two l.s. planes) are estimated using the full covariance matrix. The cell s.u.'s are taken into account individually in the estimation of s.u.'s in distances, angles and torsion angles; correlations between s.u.'s in cell parameters are only used when they are defined by crystal symmetry. An approximate (isotropic) treatment of cell s.u.'s is used for estimating s.u.'s involving l.s. planes.
Refinement. Refinement of *F*^2^ against ALL reflections. The weighted *R*-factor *wR* and goodness of fit *S* are based on *F*^2^, conventional *R*-factors *R* are based on *F*, with *F* set to zero for negative *F*^2^. The threshold expression of *F*^2^ > 2σ(*F*^2^) is used only for calculating *R*-factors(gt) *etc*. and is not relevant to the choice of reflections for refinement. *R*-factors based on *F*^2^ are statistically about twice as large as those based on *F*, and *R*- factors based on ALL data will be even larger.

### Fractional atomic coordinates and isotropic or equivalent isotropic displacement parameters (Å^2^)

**Table:**

	*x*	*y*	*z*	*U*_iso_*/*U*_eq_	
S1A	0.23713 (5)	0.62340 (4)	0.35429 (3)	0.01697 (12)	
F2A	1.06962 (13)	0.90655 (11)	0.94413 (8)	0.0227 (2)	
N4A	0.40671 (18)	0.90351 (15)	0.20331 (12)	0.0173 (3)	
N3A	0.29189 (18)	0.78138 (15)	0.21256 (12)	0.0156 (3)	
H3A	0.1939	0.7264	0.1603	0.019*	
N5A	0.50787 (17)	0.86716 (14)	0.36491 (11)	0.0143 (3)	
N6A	0.62298 (17)	0.90901 (15)	0.47624 (11)	0.0158 (3)	
C7A	0.6964 (2)	1.08701 (19)	0.33309 (15)	0.0229 (4)	
H7A1	0.6894	1.1325	0.2756	0.034*	
H7A2	0.7920	1.0658	0.3420	0.034*	
H7A3	0.7112	1.1486	0.4042	0.034*	
C8A	0.5365 (2)	0.95448 (18)	0.29812 (14)	0.0168 (4)	
C9A	0.3462 (2)	0.75585 (17)	0.31005 (14)	0.0137 (3)	
C10A	0.6362 (2)	0.80838 (17)	0.51028 (14)	0.0147 (3)	
H10A	0.5724	0.7158	0.4617	0.018*	
C11A	0.7512 (2)	0.83788 (17)	0.62529 (14)	0.0141 (3)	
C12A	0.7622 (2)	0.72535 (18)	0.65853 (14)	0.0156 (4)	
H12A	0.6968	0.6341	0.6078	0.019*	
C13A	0.8693 (2)	0.74710 (18)	0.76628 (14)	0.0164 (4)	
H13A	0.8764	0.6720	0.7886	0.020*	
C14A	0.9647 (2)	0.88390 (18)	0.83872 (14)	0.0168 (4)	
C15A	0.9588 (2)	0.99895 (18)	0.80954 (14)	0.0188 (4)	
H15A	1.0257	1.0899	0.8607	0.023*	
C16A	0.8504 (2)	0.97526 (18)	0.70181 (14)	0.0166 (4)	
H16A	0.8437	1.0510	0.6804	0.020*	
S1B	−0.10121 (5)	0.62370 (4)	0.02884 (3)	0.01602 (12)	
F2B	−0.99250 (12)	0.36565 (11)	−0.53412 (8)	0.0211 (2)	
N3B	−0.14587 (17)	0.43278 (14)	0.14306 (12)	0.0154 (3)	
H3B	−0.0436	0.4801	0.1919	0.018*	
N4B	−0.26081 (18)	0.30629 (15)	0.14576 (12)	0.0167 (3)	
N5B	−0.37235 (17)	0.36840 (14)	0.00279 (11)	0.0134 (3)	
N6B	−0.50533 (17)	0.34307 (15)	−0.09421 (11)	0.0154 (3)	
C7B	−0.5645 (2)	0.14351 (18)	0.02702 (15)	0.0201 (4)	
H7B1	−0.5561	0.0904	0.0786	0.030*	
H7B2	−0.5940	0.0854	−0.0494	0.030*	
H7B3	−0.6513	0.1734	0.0307	0.030*	
C8B	−0.3976 (2)	0.26907 (18)	0.05969 (14)	0.0154 (4)	
C9B	−0.2066 (2)	0.47588 (17)	0.05816 (13)	0.0137 (3)	
C10B	−0.4876 (2)	0.43797 (18)	−0.14289 (14)	0.0157 (4)	
H10B	−0.3874	0.5224	−0.1132	0.019*	
C11B	−0.6245 (2)	0.41410 (18)	−0.24528 (14)	0.0152 (4)	
C12B	−0.6015 (2)	0.52269 (18)	−0.29298 (14)	0.0163 (4)	
H12B	−0.5009	0.6067	−0.2589	0.020*	
C13B	−0.7250 (2)	0.50845 (18)	−0.39004 (14)	0.0165 (4)	
H13B	−0.7092	0.5812	−0.4216	0.020*	
C14B	−0.8721 (2)	0.38242 (18)	−0.43791 (13)	0.0159 (4)	
C15B	−0.9013 (2)	0.27145 (18)	−0.39334 (14)	0.0175 (4)	
H15B	−1.0024	0.1880	−0.4279	0.021*	
C16B	−0.7764 (2)	0.28784 (18)	−0.29615 (14)	0.0162 (4)	
H16B	−0.7935	0.2150	−0.2647	0.019*	

### Atomic displacement parameters (Å^2^)

**Table:**

	*U*^11^	*U*^22^	*U*^33^	*U*^12^	*U*^13^	*U*^23^
S1A	0.0138 (2)	0.0175 (2)	0.0161 (2)	0.00357 (18)	0.00235 (17)	0.00864 (17)
F2A	0.0208 (5)	0.0240 (6)	0.0141 (5)	0.0056 (5)	−0.0019 (4)	0.0066 (4)
N4A	0.0152 (7)	0.0167 (7)	0.0181 (7)	0.0047 (6)	0.0043 (6)	0.0086 (6)
N3A	0.0116 (7)	0.0161 (7)	0.0145 (7)	0.0036 (6)	0.0005 (6)	0.0059 (6)
N5A	0.0136 (7)	0.0137 (7)	0.0130 (7)	0.0050 (6)	0.0014 (6)	0.0055 (6)
N6A	0.0132 (7)	0.0180 (7)	0.0117 (7)	0.0048 (6)	0.0003 (6)	0.0052 (6)
C7A	0.0201 (9)	0.0191 (9)	0.0216 (9)	0.0021 (8)	0.0022 (8)	0.0105 (8)
C8A	0.0169 (9)	0.0182 (9)	0.0166 (8)	0.0080 (7)	0.0051 (7)	0.0089 (7)
C9A	0.0131 (8)	0.0153 (8)	0.0126 (8)	0.0074 (7)	0.0030 (7)	0.0041 (6)
C10A	0.0126 (8)	0.0150 (8)	0.0151 (8)	0.0045 (7)	0.0050 (7)	0.0045 (7)
C11A	0.0110 (8)	0.0176 (8)	0.0131 (8)	0.0051 (7)	0.0047 (7)	0.0055 (7)
C12A	0.0134 (8)	0.0152 (8)	0.0152 (8)	0.0047 (7)	0.0037 (7)	0.0035 (7)
C13A	0.0175 (9)	0.0167 (9)	0.0170 (8)	0.0083 (7)	0.0058 (7)	0.0086 (7)
C14A	0.0137 (8)	0.0233 (9)	0.0107 (8)	0.0060 (7)	0.0024 (7)	0.0069 (7)
C15A	0.0193 (9)	0.0157 (9)	0.0149 (8)	0.0034 (7)	0.0043 (7)	0.0023 (7)
C16A	0.0196 (9)	0.0161 (9)	0.0170 (8)	0.0088 (7)	0.0073 (7)	0.0084 (7)
S1B	0.0125 (2)	0.0161 (2)	0.0166 (2)	0.00421 (17)	0.00188 (17)	0.00781 (17)
F2B	0.0166 (5)	0.0258 (6)	0.0163 (5)	0.0081 (4)	−0.0012 (4)	0.0089 (4)
N3B	0.0105 (7)	0.0172 (7)	0.0151 (7)	0.0040 (6)	0.0015 (6)	0.0066 (6)
N4B	0.0151 (7)	0.0184 (7)	0.0168 (7)	0.0067 (6)	0.0050 (6)	0.0081 (6)
N5B	0.0114 (7)	0.0147 (7)	0.0118 (7)	0.0051 (6)	0.0013 (6)	0.0047 (6)
N6B	0.0135 (7)	0.0191 (7)	0.0119 (7)	0.0080 (6)	0.0009 (6)	0.0044 (6)
C7B	0.0181 (9)	0.0181 (9)	0.0191 (9)	0.0040 (7)	0.0028 (7)	0.0089 (7)
C8B	0.0184 (9)	0.0173 (9)	0.0136 (8)	0.0092 (7)	0.0070 (7)	0.0074 (7)
C9B	0.0132 (8)	0.0165 (8)	0.0127 (8)	0.0081 (7)	0.0043 (7)	0.0048 (7)
C10B	0.0131 (8)	0.0175 (9)	0.0150 (8)	0.0063 (7)	0.0032 (7)	0.0052 (7)
C11B	0.0142 (8)	0.0192 (9)	0.0135 (8)	0.0089 (7)	0.0047 (7)	0.0051 (7)
C12B	0.0129 (8)	0.0173 (9)	0.0154 (8)	0.0042 (7)	0.0044 (7)	0.0045 (7)
C13B	0.0186 (9)	0.0187 (9)	0.0161 (8)	0.0099 (7)	0.0069 (7)	0.0092 (7)
C14B	0.0126 (8)	0.0244 (9)	0.0106 (8)	0.0097 (7)	0.0018 (7)	0.0054 (7)
C15B	0.0135 (8)	0.0170 (9)	0.0176 (9)	0.0039 (7)	0.0037 (7)	0.0046 (7)
C16B	0.0164 (9)	0.0186 (9)	0.0158 (8)	0.0085 (7)	0.0060 (7)	0.0079 (7)

### Geometric parameters (Å, º)

**Table:**

S1A—C9A	1.6854 (17)	S1B—C9B	1.6855 (17)
F2A—C14A	1.3564 (19)	F2B—C14B	1.3545 (18)
N4A—C8A	1.304 (2)	N3B—C9B	1.338 (2)
N4A—N3A	1.3805 (19)	N3B—N4B	1.3785 (19)
N3A—C9A	1.341 (2)	N3B—H3B	0.8600
N3A—H3A	0.8600	N4B—C8B	1.297 (2)
N5A—C9A	1.381 (2)	N5B—C8B	1.387 (2)
N5A—C8A	1.383 (2)	N5B—C9B	1.390 (2)
N5A—N6A	1.4055 (18)	N5B—N6B	1.3935 (18)
N6A—C10A	1.282 (2)	N6B—C10B	1.277 (2)
C7A—C8A	1.482 (2)	C7B—C8B	1.484 (2)
C7A—H7A1	0.9600	C7B—H7B1	0.9600
C7A—H7A2	0.9600	C7B—H7B2	0.9600
C7A—H7A3	0.9600	C7B—H7B3	0.9600
C10A—C11A	1.468 (2)	C10B—C11B	1.463 (2)
C10A—H10A	0.9300	C10B—H10B	0.9300
C11A—C12A	1.393 (2)	C11B—C12B	1.394 (2)
C11A—C16A	1.401 (2)	C11B—C16B	1.401 (2)
C12A—C13A	1.390 (2)	C12B—C13B	1.387 (2)
C12A—H12A	0.9300	C12B—H12B	0.9300
C13A—C14A	1.378 (2)	C13B—C14B	1.377 (2)
C13A—H13A	0.9300	C13B—H13B	0.9300
C14A—C15A	1.381 (2)	C14B—C15B	1.388 (2)
C15A—C16A	1.389 (2)	C15B—C16B	1.386 (2)
C15A—H15A	0.9300	C15B—H15B	0.9300
C16A—H16A	0.9300	C16B—H16B	0.9300
			
C8A—N4A—N3A	103.69 (13)	C9B—N3B—N4B	114.48 (13)
C9A—N3A—N4A	114.26 (13)	C9B—N3B—H3B	122.8
C9A—N3A—H3A	122.9	N4B—N3B—H3B	122.8
N4A—N3A—H3A	122.9	C8B—N4B—N3B	104.14 (13)
C9A—N5A—C8A	108.36 (13)	C8B—N5B—C9B	108.24 (13)
C9A—N5A—N6A	130.31 (14)	C8B—N5B—N6B	118.40 (13)
C8A—N5A—N6A	120.57 (13)	C9B—N5B—N6B	133.34 (14)
C10A—N6A—N5A	115.20 (14)	C10B—N6B—N5B	118.87 (14)
C8A—C7A—H7A1	109.5	C8B—C7B—H7B1	109.5
C8A—C7A—H7A2	109.5	C8B—C7B—H7B2	109.5
H7A1—C7A—H7A2	109.5	H7B1—C7B—H7B2	109.5
C8A—C7A—H7A3	109.5	C8B—C7B—H7B3	109.5
H7A1—C7A—H7A3	109.5	H7B1—C7B—H7B3	109.5
H7A2—C7A—H7A3	109.5	H7B2—C7B—H7B3	109.5
N4A—C8A—N5A	110.95 (15)	N4B—C8B—N5B	110.75 (15)
N4A—C8A—C7A	126.29 (15)	N4B—C8B—C7B	126.55 (15)
N5A—C8A—C7A	122.75 (15)	N5B—C8B—C7B	122.61 (14)
N3A—C9A—N5A	102.69 (14)	N3B—C9B—N5B	102.39 (14)
N3A—C9A—S1A	127.58 (13)	N3B—C9B—S1B	127.11 (13)
N5A—C9A—S1A	129.68 (12)	N5B—C9B—S1B	130.49 (12)
N6A—C10A—C11A	120.60 (15)	N6B—C10B—C11B	120.14 (15)
N6A—C10A—H10A	119.7	N6B—C10B—H10B	119.9
C11A—C10A—H10A	119.7	C11B—C10B—H10B	119.9
C12A—C11A—C16A	119.27 (15)	C12B—C11B—C16B	119.26 (15)
C12A—C11A—C10A	118.67 (15)	C12B—C11B—C10B	117.97 (15)
C16A—C11A—C10A	122.06 (15)	C16B—C11B—C10B	122.77 (15)
C13A—C12A—C11A	121.16 (15)	C13B—C12B—C11B	121.55 (16)
C13A—C12A—H12A	119.4	C13B—C12B—H12B	119.2
C11A—C12A—H12A	119.4	C11B—C12B—H12B	119.2
C14A—C13A—C12A	117.62 (15)	C14B—C13B—C12B	117.43 (16)
C14A—C13A—H13A	121.2	C14B—C13B—H13B	121.3
C12A—C13A—H13A	121.2	C12B—C13B—H13B	121.3
F2A—C14A—C13A	118.21 (15)	F2B—C14B—C13B	118.19 (15)
F2A—C14A—C15A	118.41 (15)	F2B—C14B—C15B	118.62 (15)
C13A—C14A—C15A	123.38 (15)	C13B—C14B—C15B	123.18 (15)
C14A—C15A—C16A	118.25 (16)	C16B—C15B—C14B	118.52 (16)
C14A—C15A—H15A	120.9	C16B—C15B—H15B	120.7
C16A—C15A—H15A	120.9	C14B—C15B—H15B	120.7
C15A—C16A—C11A	120.32 (16)	C15B—C16B—C11B	120.06 (16)
C15A—C16A—H16A	119.8	C15B—C16B—H16B	120.0
C11A—C16A—H16A	119.8	C11B—C16B—H16B	120.0
			
C8A—N4A—N3A—C9A	0.33 (18)	C9B—N3B—N4B—C8B	0.09 (18)
C9A—N5A—N6A—C10A	42.7 (2)	C8B—N5B—N6B—C10B	175.50 (14)
C8A—N5A—N6A—C10A	−148.60 (15)	C9B—N5B—N6B—C10B	−6.8 (3)
N3A—N4A—C8A—N5A	1.13 (18)	N3B—N4B—C8B—N5B	−0.02 (18)
N3A—N4A—C8A—C7A	−179.27 (17)	N3B—N4B—C8B—C7B	−176.50 (16)
C9A—N5A—C8A—N4A	−2.17 (19)	C9B—N5B—C8B—N4B	−0.05 (19)
N6A—N5A—C8A—N4A	−173.13 (14)	N6B—N5B—C8B—N4B	178.23 (13)
C9A—N5A—C8A—C7A	178.21 (16)	C9B—N5B—C8B—C7B	176.60 (15)
N6A—N5A—C8A—C7A	7.2 (2)	N6B—N5B—C8B—C7B	−5.1 (2)
N4A—N3A—C9A—N5A	−1.58 (18)	N4B—N3B—C9B—N5B	−0.11 (17)
N4A—N3A—C9A—S1A	176.05 (12)	N4B—N3B—C9B—S1B	−179.39 (12)
C8A—N5A—C9A—N3A	2.17 (17)	C8B—N5B—C9B—N3B	0.09 (17)
N6A—N5A—C9A—N3A	171.95 (15)	N6B—N5B—C9B—N3B	−177.83 (15)
C8A—N5A—C9A—S1A	−175.39 (13)	C8B—N5B—C9B—S1B	179.34 (13)
N6A—N5A—C9A—S1A	−5.6 (3)	N6B—N5B—C9B—S1B	1.4 (3)
N5A—N6A—C10A—C11A	−179.52 (13)	N5B—N6B—C10B—C11B	179.62 (14)
N6A—C10A—C11A—C12A	−179.68 (15)	N6B—C10B—C11B—C12B	177.84 (15)
N6A—C10A—C11A—C16A	−0.1 (2)	N6B—C10B—C11B—C16B	−1.8 (3)
C16A—C11A—C12A—C13A	0.2 (2)	C16B—C11B—C12B—C13B	−0.4 (2)
C10A—C11A—C12A—C13A	179.80 (15)	C10B—C11B—C12B—C13B	179.94 (15)
C11A—C12A—C13A—C14A	−0.3 (2)	C11B—C12B—C13B—C14B	0.0 (2)
C12A—C13A—C14A—F2A	179.92 (14)	C12B—C13B—C14B—F2B	−178.64 (14)
C12A—C13A—C14A—C15A	0.0 (3)	C12B—C13B—C14B—C15B	0.3 (3)
F2A—C14A—C15A—C16A	−179.57 (14)	F2B—C14B—C15B—C16B	178.77 (14)
C13A—C14A—C15A—C16A	0.4 (3)	C13B—C14B—C15B—C16B	−0.2 (3)
C14A—C15A—C16A—C11A	−0.4 (2)	C14B—C15B—C16B—C11B	−0.2 (2)
C12A—C11A—C16A—C15A	0.1 (2)	C12B—C11B—C16B—C15B	0.5 (2)
C10A—C11A—C16A—C15A	−179.41 (15)	C10B—C11B—C16B—C15B	−179.81 (16)

### Hydrogen-bond geometry (Å, º)

**Table:**

*D*—H···*A*	*D*—H	H···*A*	*D*···*A*	*D*—H···*A*
N3*A*—H3*A*···S1*B*	0.86	2.45	3.2840 (18)	164
N3*B*—H3*B*···S1*A*	0.86	2.51	3.3691 (18)	172
